# Buried bumper syndrome: a case report of an early PEG gastropexy-associated complication in a patient with gastric volvulus

**DOI:** 10.1093/jscr/rjab261

**Published:** 2021-07-05

**Authors:** Amenah Dhannoon, Maha AlKhattab, Rishabh Sehgal, Chris G Collins

**Affiliations:** Department of Surgery, University Hospital Galway, Galway, Ireland; Department of Surgery, University Hospital Galway, Galway, Ireland; Department of Surgery, University Hospital Galway, Galway, Ireland; Department of Surgery, University Hospital Galway, Galway, Ireland; Academic Department of Surgery, National University of Ireland Galway, Galway, Ireland

## Abstract

Buried bumper syndrome (BBS) is a rare complication associated with percutaneous endoscopic gastrostomy (PEG) tubes. It develops when the internal bumper migrates through the gastric wall, lodging anywhere along the gastrostomy tract leading to overgrowth of gastric mucosa thereby encasing the tube. BBS can lead to bleeding, perforation, peritonitis and intra-abdominal sepsis. Our case is a 71-year-old female presenting with tenderness, erythema and purulent discharge at the PEG tube site 2-weeks post-insertion. Computer tomography scan demonstrated the PEG had dislodged with the internal bumper in the subcutaneous tissue and the distal tip lying within the tract beyond the stomach wall. The PEG was removed by simple external traction. The patient clinically improved and discharged home on day three.

Although BBS usually occurs late post-PEG insertion, it can also occur acutely. Preventative measures should be adopted at ward-level and emphasized with appropriate PEG tube care information provided to patients to avoid and recognize such complication.

## INTRODUCTION

Percutaneous endoscopic gastrostomy (PEG) tube insertion has been increasingly used since the 1980s as a route for enteral feeding and nutritional support [1]. They can also be used for anchoring the stomach to the anterior abdominal wall during a gastropexy to prevent hiatus hernia and gastric volvulus. This technique is effective in patients who are at high risk for paraesophageal hernia surgery [2].

**Figure 1:**
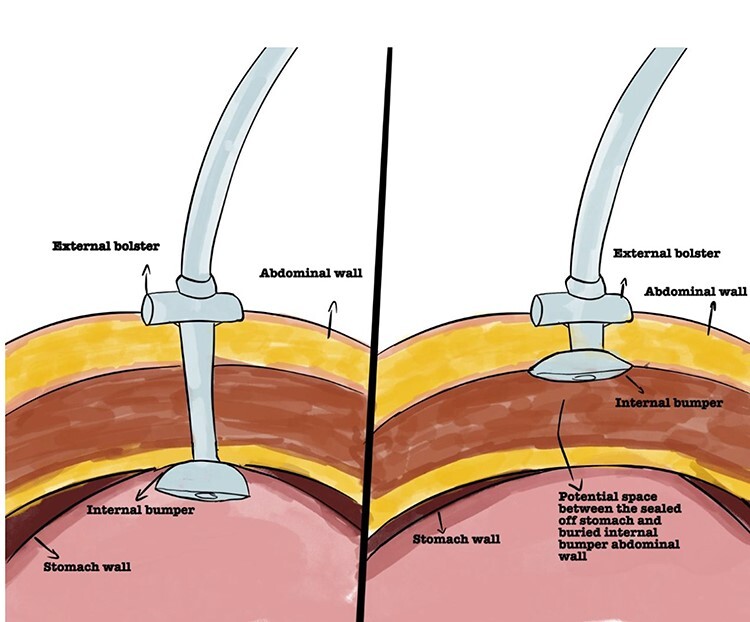
Representation of normal PEG positioning (right) and BBS (left).

Even though placement of a PEG tube is considered safe, it carries potential risks and complications ranging between 0.4 and 22.5% [3]. Early complications include PEG dysfunction, dislodgement, infection and aspiration. Buried bumper syndrome (BBS) occurs in 0.3% to 2.5% post PEG insertion. It develops when the internal bumper of the PEG migrates through the gastric wall, lodging anywhere along the gastrostomy tract leading to the mucosa to partially or completely grow over it (Fig. 1) [4, 5]. This can lead to the inability to flush the PEG tube, blockage and leakage of gastric contents causing skin irritation and infection [4, 6]. BBS is generally considered a delayed complication typically occurring between 2 months up and 7 years post-PEG insertion. However, Lee *et al*. [6] demonstrated the development of BBS at Day 8 [6]. If left unrecognized, BBS can lead to the development of gastric bleeding, perforation, peritonitis, abdominal wall collections and sepsis [7, 8]. Therefore, prompt recognition and diagnosis of this condition is paramount to instigate timely management.

We highlight a case of BBS that developed acutely following PEG insertion.

## CASE REPORT

A 71-year-old female presented to the Emergency Department (ED) complaining of abdominal pain and peristomal tenderness with associated erythema and purulent discharge from her PEG tube gastropexy site. The PEG tube had been inserted endoscopically under general anaesthetic 2 weeks earlier for the prevention of previous repeated episodes of gastric volvulus and diaphragmatic herniation. Her medical history was significant for hypertension and hypothyroidism. Prior to presentation to the ED, she had been treated for peristomal cellulitis with a 5-day course of oral flucloxacillin (250 mg qds), which lead to only modest clinical improvement. At the time of ED presentation, the pain and discharge had increased significantly.

Laboratory examination demonstrated white cell count 4.9 cells/μl and an elevated C-reactive protein level, 13.6 mg/L. On examination, the PEG tube showed signs of infection and inflammation secondary to peristomal leakage.

**Figure 2:**
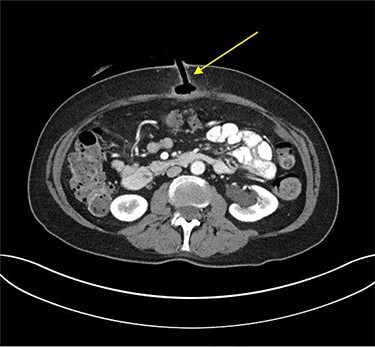
Transverse plane of CT of abdomen and pelvis illustrating the migration of the internal bumper of the PEG and the sealing of gastric mucosa as indicated by the yellow arrow.

Erect plain chest radiograph was normal. Computed tomography (CT) imaging of the abdomen and pelvis demonstrated evidence of PEG tube dislodgement with the internal bumper positioned in the subcutaneous tissue and the distal tip of the tube in the tract beyond the stomach wall, consistent with a diagnosis of BBS (Fig. 2).

As the primary indication for the PEG placement was gastropexy, it was safely removed in the ED by using simple external traction. A wound bag was placed over the wound site temporarily. The patient was commenced on broad-spectrum antibiotics (Co-amoxiclav 1.2 g IV tds). The patient’s pain and erythema reduced, with down-trending inflammatory markers. The PEG opening sealed off with the use of standard dressings. The patient was discharged home on Day 3. She made an uneventful recovery and was symptom free when reviewed at her 2-week outpatient clinic appointment.

## DISCUSSION

The BBS is considered a delayed complication of PEG tube insertion that can lead to potential severe complications because of the delayed diagnosis or left untreated. The causative factors for the development of BBS are multifactorial. Firstly, the mechanical pressure of the PEG exerting onto the stomach wall can lead to pressure necrosis secondary to mucosal ischaemia. This weakens the layers in the gastric wall thereby facilitating the migration of the internal stump towards the skin [9]. Furthermore, surgical technical factors such as excessive tightness of the external bumper during PEG tube insertion and the material of the tube may contribute to the development of BBS. Moreover, PEG gastropexies can alter the tension from upward migration of the stomach to its previous location in the thoracic cavity as well as the negative intra-pleural pressure taken together can contribute to the development of BBS.

Patient factors can also contribute due to inadequate handling techniques, poor hygiene, excessive external pulling and comorbidities such as obesity, chronic cough, agitation and poor cooperation [3, 9]. However, this was not the case in our patient. Table 1 summarizes the potential causative factors for BBS.

**Table 1:** Summary of causative factors for the development of BBS

Physiological factors	PEG tube factors	Patient factors
• Excessive secretion of gastric secretion including hydrochloric acid and pepsin leading to physical alteration	• Excessive tightness on the external bumper • Material and the size of the tube	• Poor handling technique • Excessive pulling • Comorbidities (high BMI, chronic cough, Alzheimer or dementia)

BMI, body mass index.

There are several management options for BBS. These include conservative, endoscopic and surgical modalities. The first approach is usually reserved for patients who are deemed high operative risk and are considered poor surgical candidates. For such patients the buried part of the PEG tube is released by external traction or by a skin incision performed under local anaesthesia [10, 11]. It is hoped that the inflammatory response at the internal gastrostomy site would be adequate to maintain the iatrogenic fistulous tract between the stomach and abdominal wall. An interval CT scan should be performed to monitor for tract maturity.

Endoscopic methods have been described in the literature whereby the mucosa is dissected using a needle knife together with a push-pull T-technique. However, this method can be complicated by bleeding and perforation [4]. Wolpert *et al.* [12] described pulling the bumper from the gastric wall towards the lumen using an endoscopic L-shaped cutting wire such as a HookKnife (Olympus Endotherapy) to dissect the overgrown gastric mucosa from over the bumper using diathermy [12].

Surgical management options include performing more invasive procedures under general anaesthesia such as laparoscopy and laparotomy. Even though laparotomy is reserved for BBS cases complicated by abscess formation [13], the laparoscopic approach has been shown to be a safe approach allowing the removal and simultaneous reinsertion of a new PEG tube [3, 14].

In the absence of defined management guidelines, it is imperative for all allied healthcare personnel that are involved in the patients’ care to ensure both patients and their families are provided with proper education and post PEG care packages prior to discharge to minimize complications such as BBS. Care instructions should be provided and emphasized by the healthcare staff involved. These include information regarding proper handwashing technique prior to manipulating the PEG tube, cleaning methods and advancing and rotating the tube full cycle before reapplying the external fixator [15].

In conclusion, while BBS is known to be a rare late complication of PEG tube insertion, it can occur in the acute phase as demonstrated in this case report. It is important that this is considered in the differential diagnosis of an apparently infected PEG site. Prompt removal of the PEG tube is indicated to prevent further complications. Management can be conservative, endoscopic or surgical depending on the clinical presentation. Preventative measures can be adopted at ward-level and should be recommended upon discharge to the community.
